# The Role of Complement Component C3 Activation in the Clinical Presentation and Prognosis of IgA Nephropathy—A National Study in Children

**DOI:** 10.3390/jcm10194405

**Published:** 2021-09-26

**Authors:** Małgorzata Mizerska-Wasiak, Agnieszka Such-Gruchot, Karolina Cichoń-Kawa, Agnieszka Turczyn, Jadwiga Małdyk, Monika Miklaszewska, Dorota Drożdż, Agnieszka Firszt-Adamczyk, Roman Stankiewicz, Agnieszka Rybi-Szumińska, Anna Wasilewska, Maria Szczepańska, Beata Bieniaś, Przemysław Sikora, Agnieszka Pukajło-Marczyk, Danuta Zwolińska, Monika Pawlak-Bratkowska, Marcin Tkaczyk, Jacek Zachwieja, Magdalena Drożyńska-Duklas, Aleksandra Żurowska, Katarzyna Gadomska-Prokop, Ryszard Grenda, Małgorzata Pańczyk-Tomaszewska

**Affiliations:** 1 Department of Pediatrics and Nephrology, Medical University of Warsaw, 02-091 Warsaw, Poland; agnieszkasuch.g@gmail.com (A.S.-G.); karolina.cichon@yahoo.com (K.C.-K.); agamosionek@gmail.com (A.T.); mpanczyk1@wum.edu.pl (M.P.-T.); 2 Department of Pathology, Medical University of Warsaw, 02-091 Warsaw, Poland; jagusia.maldyk@wp.pl; 3 Department of Pediatric Nephrology and Hypertension, Jagiellonian University Medical College, 30-663 Cracow, Poland; mmiklasz@wp.pl (M.M.); dorota_drozdz@poczta.onet.pl (D.D.); 4 Department of Pediatrics and Nephrology, Ludwik Rydygier Hospital, 87-100 Torun, Poland; afirszt1@wp.pl (A.F.-A.); rstan@wp.pl (R.S.); 5 Department of Pediatrics and Nephrology, Medical University of Bialystok, 15-089 Bialystok, Poland; arybiszuminska@gmail.com (A.R.-S.); annwasil@interia.pl (A.W.); 6 Department of Pediatrics, SMDZ in Zabrze, Silesian Medical University, 41-808 Zabrze, Poland; szczep57@poczta.onet.pl; 7 Department of Pediatric Nephrology, Medical University of Lublin, 20-059 Lublin, Poland; beatabienias@umlub.pl (B.B.); sikoraprzem@hotmail.com (P.S.); 8 Department of Pediatric Nephrology, Wroclaw Medical University, 50-367 Wroclaw, Poland; pukajlo@o2.pl (A.P.-M.); danuta.zwolinska@umed.wroc.pl (D.Z.); 9 Department of Pediatrics, Immunology and Nephrology, Polish Mothers Memorial Hospital Research Institute, 93-338 Lodz, Poland; monika.bratkowska@gmail.com (M.P.-B.); marcin.tkaczyk45@gmail.com (M.T.); 10 Department of Pediatric Nephrology and Dialysis, Medical University of Poznan, 61-701 Poznan, Poland; j.zachwieja@mp.pl; 11 Department of Pediatrics, Nephrology and Hypertension, Medical University of Gdansk, 80-210 Gdansk, Poland; magdalena.drozynska-duklas@gumed.edu.pl (M.D.-D.); aleksandra.zurowska@gumed.edu.pl (A.Ż.); 12 Department of Nephrology, Kidney Transplantation and Hypertension, Children’s Memorial Health Institute, 04-730 Warsaw, Poland; k.gadomska@czd.pl (K.G.-P.); r.grenda@ipczd.pl (R.G.)

**Keywords:** IgA nephropathy, complement C3, children

## Abstract

The aim of the study was to evaluate the influence of the intensity of mesangial C3 deposits in kidney biopsy and the serum C3 level on the clinical course and outcomes of IgAN in children. The study included 148 children from the Polish Pediatric IgAN Registry, diagnosed based on kidney biopsy. Proteinuria, creatinine, IgA, C3 were evaluated twice in the study group, at baseline and the end of follow-up. Kidney biopsy was categorized using the Oxford classification, with a calculation of the MEST-C score. The intensity of IgA and C3 deposits were rated from 0 to +4 in immunofluorescence microscopy. The intensity of mesangial C3 > +1 deposits in kidney biopsy has an effect on renal survival with normal GFR in children with IgAN. A reduced serum C3 level has not been a prognostic factor in children but perhaps this finding should be confirmed in a larger group of children.

## 1. Introduction

IgA nephropathy (IgAN) or Berger’s disease is the most common chronic glomerulonephritis worldwide [1].

In Europe, it is diagnosed in 20% of kidney biopsies performed in childhood [2]. The condition is one of the major causes of end-stage renal disease (ESRD) which develops in 20–40% of patients at 20 years after the diagnosis [3].

The clinical presentation of IgAN may vary, reflecting a wide range of histological findings, from no changes on light microscopy to severe necrotizing lesions with crescents [4]. Clinically, IgAN manifests with persistent or periodic erythrocyturia, either isolated or with concomitant proteinuria of varying severity, sometimes accompanied by hypertension.

The gold standard for the diagnosis of IgAN is the evaluation of a kidney biopsy specimen. The disease is diagnosed based on the predominant IgA deposits on histopathological evaluation. The deposits may also include immunoglobulins M or G. In 90% of cases, the complement component C3 is also identified in the kidney biopsy specimen [3,5]. The Oxford classification (MEST-C) used to evaluate kidney biopsies allows the assessment of risk factors for future renal failure [6].

Proteinuria, reduction of the glomerular filtration rate (GFR), hypertension, old age, male sex and the absence of macroscopic hematuria are independent predictors of a poor outcome of the disease [7,8,9,10,11].

The pathophysiology of the disease is not entirely understood. According to the “four-hits” theory, the initial underlying insult is overproduction of abnormal, galactose-deficient immunoglobulin A1 (GdIgA1) which forms polymers (first hit). Then, specific IgA and/or IgG antibodies against the abnormal IgA1 are produced (second hit), combining and forming circulating immune complexes (third hit). These complexes accumulate in the renal mesangium, inducing a chronic inflammatory response by increased cytokine and growth factor production, which leads to cellular proliferation and mesangial matrix expansion (fourth hit) [2,12,13]. Chronic inflammation results in renal parenchymal fibrosis and progressive renal failure.

A key role in the pathogenesis and progression of IgAN is played by the complement system activation [3,14,15]. IgAN-associated processes involve the alternative and lectin pathways. The processes associated with complement activation likely occur systemically, in the circulating IgA-containing immune complexes and the glomeruli [16].

In the immune system, the ultimate effect of the complement system activation is the formation of C5b-9 sequence (membrane attacking complex, MAC) which perforates the cell membranes of pathogens. Mesangial MAC deposits are commonly observed in IgAN, and its presence is identified by the detection of C9 neoantigen corresponding to the C5b-9. Urinary excretion of the soluble form of MAC was found to be increased in patients with IgAN, likely due to complement activation in the urinary space [16].

The aim of the study was to evaluate the influence of the severity of mesangial C3 deposits in kidney biopsy specimens and the serum C3 level on the clinical course and outcomes of IgAN in children.

## 2. Material and Methods

The study included 148 children (91 boys and 56 girls) from the 166 patients included in the Polish Pediatric IgAN Registry. The patients included in the study fulfilled the following inclusion criteria: IgAN diagnosed based on kidney biopsy with evaluation by light microscopy and immunofluorescence. Patients without complete clinical and histopathological data, with the glomerular number < 8, with secondary IgAN and IgA vasculitis nephritis (IgAVN, Henoch-Schönlein nephritis) were excluded from the study.

Proteinuria and serum levels of albumin, creatinine, IgA, C3 and C4 were evaluated twice in the study group, at baseline and the end of follow-up.

Nephrotic range proteinuria was defined as ≥50 mg/kg/d, and non-nephrotic range proteinuria as <50 mg/kg/d, and urinary protein was measured by the Exton method. Serum creatinine level, expressed in mg/dL, was measured by the dry chemistry method (Vitro, Ortho Clinical Diagnostic). GFR (mL/min/1.73 m^2^) was estimated using the Schwartz formula. Immunoglobulin A and complement component C3 and C4 serum levels were measured by the nephelometric method in 5 centers and by the turbidimetric method in 3 centers. The use of two different methods for assaying immunoglobulin A and C3 and C4 were related to the retrospective nature of the study conducted in various centers. Referring to studies by Denham et al. which indicate good agreement between methods in determining protein levels, including IgA and C3, we considered it as a limitation of the study, but the age-related reference ranges did not differ significantly between the centers [17].

A diagnostic kidney biopsy was performed on all children in the study group.

The specimens from each kidney biopsy were routinely evaluated using light microscopy, immunofluorescence and electron microscopy by at least two pathologists.

Kidney biopsy specimens were routinely evaluated by light microscopy and immunofluorescence, and categorized using the Oxford classification, with a calculation of the MEST-C score (1—present, 0—absent; M—mesangial hypercellularity; E—endocapillary hypercellularity; S—segmental sclerosis/adhesion; T—tubular atrophy/interstitial fibrosis T0 0–25%, T1 26–50%, T2 > 50%; C—crescents, C0 0%, C1 0–25%, C2 > 25%; with the overall score calculated as the sum of M, E, S, T and C).

When evaluated by immunofluorescence microscopy, the intensity of IgA and C3 deposits were rated from 0 to +4.

The patients received renoprotective therapy (angiotensin-converting enzyme inhibitor [ACEI]/angiotensin receptor blocker [ARB]), glucocorticosteroids (Encorton) or immunosuppressive drugs such as azathioprine, cyclophosphamide, cyclosporin A and mycophenolate mofetil. Drug treatment was categorized as I—immunosuppression, S—steroids, R—renoprotection.

The study endpoint was an abnormal glomerular filtration rate (eGFR <90 mL/min).

The study was approved by the Bioethics Committee at the Medical University of Warsaw (No. KB/147/2017). Informed consent for study participation was obtained from the legal guardians of the study participants.

**Figure 1:**
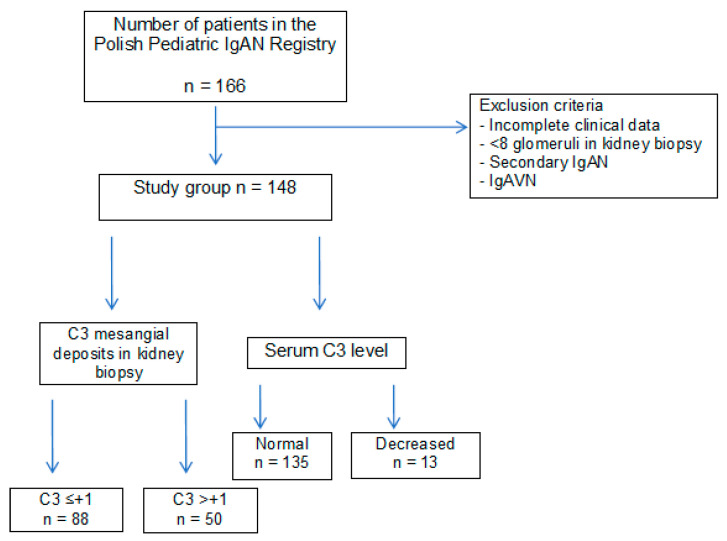
Flow diagram of the study.

Flow diagram of the study is shown in Figure 1.

### Statistical Analysis

Statistical analysis was performed using the Dell Statistica 13.0 PL software. The results were expressed as the mean values and standard deviation for normally distributed variables and as the median and range for non-normally distributed variables. The normality of distributions was evaluated using the Lilliefors and Shapiro-Wilk tests. The significance of differences between the mean values was evaluated using the ANOVA for normally distributed variables and the Kruskal-Wallis test for non-normally distributed variables. The significance of differences between groups was determined using the Student *t*-test (for normally distributed variables) and the Mann-Whitney test (for non-normally distributed variables). To evaluate differences between baseline and follow-up values, the Student *t-* test and the Wilcoxon test were used (for normally and non-normally distributed variables, respectively). *p* < 0.05 was considered statistically significant. The Kaplan-Meier and Cox regression analyses were performed to calculate renal survival.

## 3. Results

The characteristics of the study group are shown in Table 1.

The mean age at the diagnosis of IgAN was 11 ± 4.29 years. Boys and girls comprised 61.47% and 38.53% of the study group, respectively. A kidney biopsy was performed on average 1.2 ± 1.77 years since the initial symptoms, and the mean duration of follow-up was 45 ± 30.75 months.

At baseline, the mean proteinuria was 44.57 ± 120.04 mg/kg/d, creatinine level was 0.73 ± 0.33 mg/dL, and GFR was 96.75 ± 33.56 mL/min/1.73 m^2^. At the end of follow-up, the mean proteinuria was 10.11 ± 35.84 mg/kg/d, creatinine level was 0.71 ± 0.22 mg/dL and GFR was 100.65 ± 22.86 mL/min/1.73 m^2^.

At baseline, an elevated IgA level was noted in 73 patients (49.32%), and reduced C3 and C4 levels in 13 (8.78%) and 17 patients (11.49%), respectively. At the end of follow-up, an elevated IgA level was noted in 47 patients (31.76%), reduced C3 level in 12 (8.10%) and reduced C4 level in 26 patients (17.57%).

GFR was <90 mL/min in 58 (39.19%) children at baseline and in 46 (31.08%) children at the end of follow-up.

Regarding to the evaluation by the Oxford classification, mesangial hypercellularity (M1) was present in 81.76% of patients in the study group, endocapillary hypercellularity (E1) in 23.65%, segmental sclerosis (S1) in 28.39%, interstitial fibrosis/tubular atrophy (T1/2) in 18.24% and cellular/fibrocellular crescents (C1/2) in 27.7%.

The study group was categorized based on the presence of C3 deposits in kidney biopsy specimens. Low severity of C3 deposits was defined as C3 ≤ +1, and high severity as C3 > +1. Depending on the presence of C3 deposits in kidney biopsy specimens, the patients were divided into two groups: group A—C3 ≤ 1, group B—C3 > 1. The duration of follow-up was 4.19 ± 3.05 years in group A, and 2.91 ± 2.46 years in group B.

The clinical characteristics of the study patients divided into two groups based on the severity of C3 deposits are shown in Table 2.

No differences between group A (*n* = 98) and group B (*n* = 50) were found regarding to proteinuria and GFR at baseline and the end of follow-up. Serum creatinine level and severity of IgA and C3 deposits in kidney biopsy were significantly higher in group B (*p* < 0.01).

There were no significant differences between the two groups regarding to the overall MEST-C score.

Renoprotective treatment was used in 58 (59.2%) patients in group A and 21 (42.0%) patients in group B. Glucocorticosteroids were used in 13 (13.3%) patients in group A and 11 (22.0%) patients in group B. Immunosuppressive therapy was administered in 27 (27.6%) patients in group A and 17 (34.0%) patients in group B. Regarding to the drug treatment used, there was a significant difference only in renoprotective treatment between the two groups, there were no significant differences in glucocorticosteroids and immunosuppressive therapy.

There was no difference in the mean GFR at the end of follow-up between patients in groups A and B, as well the percentages of patients with GFR >90 and <90 mL/min (*p* = 0.08).

**Figure 2:**
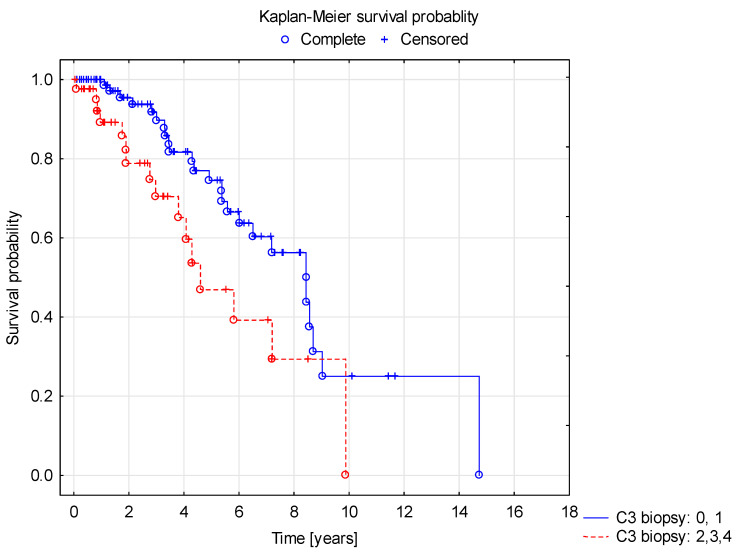
Shorter renal survival with normal GFR in Group B (intensity of C3 deposits = +2, +3, +4) vs. Group A (intensity of C3 deposits = 0, +1).

**Figure 3:**
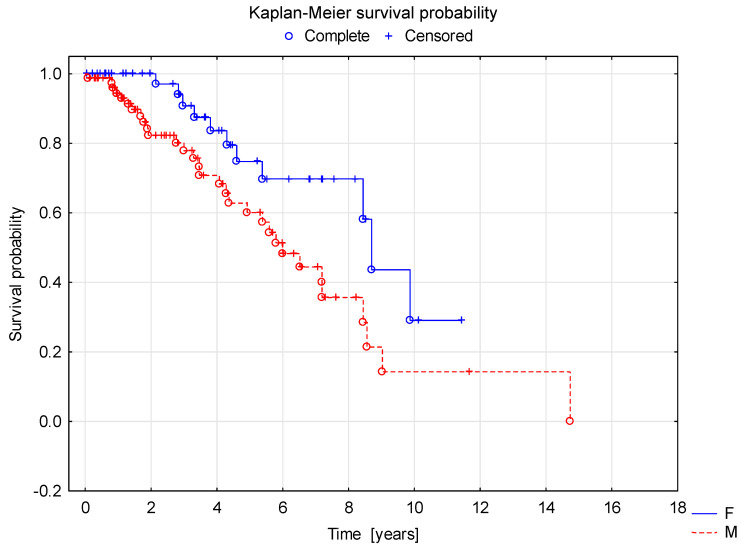
Shorter renal survival with normal GFR in males vs. females.

**Figure 4:**
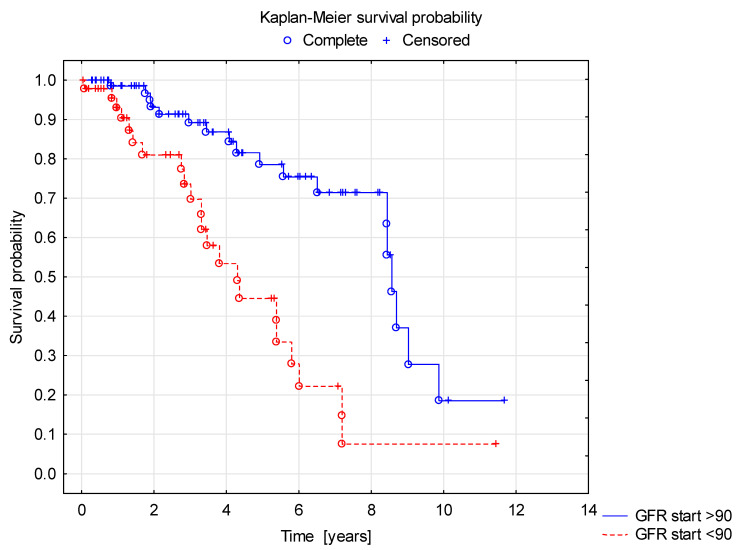
Shorter renal survival with normal GFR in patients with reduced GFR (<90 mL/min) at the time of the diagnosis.

Survival curve analysis using the Cox proportional hazard model showed a shorter duration of renal survival with normal GFR in children in group B (C3 >1 in kidney biopsy) compared to group A (C3 ≤ 1) (Figure 2). In the survival curve analysis, factors affecting longer renal survival with normal GFR included female gender (F > M, Figure 3), older age at the diagnosis and normal GFR at the onset of the disease (Figure 4).

The study group was also divided regarding to the MEST-C score (group I—MEST-C score ≤ 1, group II—MEST-C score > 1). The clinical characteristics of patients in these two groups are shown in Table 3. There were no significant differences between groups I and II regarding to albumin, C3 and C4 levels at baseline and the end of follow-up, and the severity of IgA, IgG and IgM deposits in kidney biopsy. Serum creatinine level at baseline was significantly higher in group II (*p* < 0.001), as was IgA level (*p* < 0.01). A significant difference was also found in GFR at baseline (group I > II; *p* < 0.01). At the end of follow-up, a significant difference was noted only for proteinuria which was higher in group II (*p* < 0.05).

The study group was also divided based on serum C3 level (Group 1—serum C3 level below the reference range, Group 2—serum C3 level within the reference range). The clinical characteristics of patients in these two groups are shown in Table 4. No significant differences between Groups 1 and 2 were found regarding to the severity of proteinuria, GFR and creatinine, albumin and IgA levels at baseline and at the end of follow-up, as well as the intensity of IgA deposits in renal biopsy. Serum C4 level at baseline was significantly higher in Group 2 (*p* = 0.01) but no significant difference in C4 level was found between the groups at the end of follow-up.

Survival curve analysis using the Cox proportional hazard model showed no difference in renal survival with normal GFR between groups with normal and reduced serum C3 levels at baseline.

## 4. Discussion

In this retrospective study, we performed a detailed evaluation of the importance of C3 in kidney biopsy specimens and serum for the outcomes of IgAN in children. We found that the presence of >+1 C3 deposits in kidney biopsy is a predictor of worsening renal function (GFR < 90 mL/min) in this group of children, which is the first such study in a large sample collected throughout a European country. However, we did not show similar importance of a reduced serum C3 level at the onset of the disease.

In the study population, C3 deposits in kidney biopsy, mostly rated at +1 or +2, were identified in 66% of patients, which is a rate similar to that reported in an adult population studied by Wu et al. [18].

In patients with IgAN, immune complexes may activate the alternative and lectin pathways of the complement system and initiate inflammation [13,15,19]. Recent studies in adult patients show that the severity of C3 deposits in kidney biopsy and reduced serum C3 level may affect long-term renal outcomes [3,20,21,22].

In our study from 2015, we evaluated the usefulness of serum Immunoglobulin A/complement factor 3 (IgA/C3) ratio for predicting the severity of histological lesions in kidney biopsy children with IgA nephropathy. We found positive correlations between the IgA/C3 ratio and proteinuria, serum creatinine and serum IgA level. We also determined that the higher the MEST score the higher the IgA/C3 ratio. We also determined the optimal cutoff values of IgA/C3 serum ratio for specific MEST score [23].

In the study by Caliskan et al. in 111 adult patients with IgAN, C3 deposits > +1 were found to be a prognostic factor for the development of chronic kidney disease (CKD) stage G5 or reduction in GFR by ≥50% compared to the baseline [20].

We also confirmed the importance of C3 standing > +1 as an adverse prognostic factor for renal survival in children but in our study, the endpoint was GFR < 90 mL/min, and thus we demonstrated the prognostic significance of C3 deposits for CKD from stage G2 onwards, which is a novel finding, and these observations were made in children, which also contrasts to the conclusions from the studies in adult patients with IgAN [20].

In the study by Kim and Koo in 66 adult patients with IgAN, a prognostically adverse effect of C3 deposits > +1 for the development of ESRD or doubling of serum creatinine level was also shown, and this study also showed an effect of reduced serum C3 level on the renal outcomes, although its predictive value was lower than that of the urinary protein to creatinine ratio [22]. In our study, we were unable to confirm the effect of reduced serum C3 level on renal survival with normal GFR but this may have been related to a low number of children (*n* = 13, or 8.78%) with reduced serum C3 level at baseline. This finding needs to be replicated in a larger patient sample.

An additional, though already previously known finding of our study is confirmation of an adverse effect of male gender and reduced GFR at baseline on long-term renal outcomes [8,9,10,11].

Among children with C3 deposits > +1 and those with a reduced serum C3 level at baseline, we did not find significant differences in the MEST-C score, similarly to the study by Kim et al. who did not find significant differences in the rates of M, E, S and T between groups with C3 deposits > +1 and ≤ +1 [22].

In addition, we found a reduced serum C3 level at the end of follow-up in 10 patients, of whom 3 showed a reduced serum C3 level at baseline. Of these, only one patient had GFR < 90 mL/min at the end of follow-up, which might also confirm no prognostic significance of a reduced serum C3 level at baseline in children, but again, these patient groups were too small to allow any definitive conclusions.

## 5. Conclusions

The severity of mesangial C3 deposits in kidney biopsy rated > +1 has an effect on renal survival with normal GFR in children with IgAN. A reduced serum C3 level has not been a prognostic factor in this group of children but perhaps this finding should be confirmed in a larger group of children.

## Figures and Tables

**Table 1:** Characteristics of the study group.

Parameter	IgAN (*n* = 148)
Age at disease onset (years)	11.6 ± 4.29
Gender (M/F)	91/57
Time to biopsy (years)	1.2 ± 1.77
Proteinuria (mg/kg/d)	14.0 (0.0–967.0)
GFR (mL/min/1.73 m^2^)	95.75 ± 33.56
Creatinine (mg/dL)	0.73 ± 0.33
Albumin (mg/dL)	3.84 ± 0.86
IgA (mg/dL)	275.91 ± 134.45
C3 (mg/dL)	118.12 ± 29.54
C4 (mg/dL)	23.55 ± 8.25
IgA deposits in kidney biopsy/number of patients (*n*%)	148 (100%)
C3 deposits in kidney biopsy (*n*%)	98 (66.2%)
Duration of follow-up (years)	3.75 ± 2.90
Age at FU (years)	15.26 ± 3.84
Proteinuria at FU (mg/kg/d)	0.0 (0–370.0)
GFR at FU (mL/min/1.73 m^2^)	100.65 ± 22.86
Creatinine at FU (mg/dL)	0.71 ± 0.22
Albumin at FU (mg/dL)	4.3 ± 0.48
IgA at FU (mg/dL)	257.26 ± 122.38
C3 at FU (mg/dL)	106.61 ± 24.87
C4 at FU (mg/dL)	22.61 ± 14.67
Treatment:	
ACEI/ARB/none	43.24% (*n* = 64)
Glucocorticosteroids alone	29.73% (*n* = 44)
Immunosuppression + glucocorticosteroids	16.21% (*n* = 24)

ACEI—angiotensin-converting enzyme inhibitor; ARB—angiotensin receptor blocker; FU—end of follow-up; *n*—number of patients.

**Table 2:** Clinical characteristics of the patient groups based on the severity of C3 deposits.

	Group A (*n* = 98) C3 ≤ +1	Group B (*n* = 50) C3 > +1	*p*
Age at diagnosis (years)	11.26 ± 4.39	12.34 ± 4.06	NS
Proteinuria at baseline (mg/kg/d)	14.0 (0–968)	15.0 (0–202)	NS
Creatinine at baseline (mg/dL)	0.72 ± 0.36	0.76 ± 0.25	NS
GFR at baseline (mL/min)	96.45 ± 35.26	94.44 ± 30.96	NS
Time to biopsy (years)	1.33 ± 1.96 median 0.52	0.92 ± 13.31 median 0.31	NS (*p* = 0.06)
Intensity of IgA deposits (*n*%)			
+1	47 46.5 %	1 2.0 %	*p* < 0.00001
+2	23 23.2	6 12.0	
+3	20 20.2	23 46.0	
+4	9 9.1	20 40.0	
Overall MEST-C score	1.61 ± 1.08	1.67 ±1.04	NS
M1 *n* (%)	79 (80.6%)	42 (84.0%)	
E1 *n* (%)	25 (25.5%)	10 (20.0%)	
S1 *n* (%)	24 (24.5%)	18 (36.0%)	
T1-2 *n* (%)	17 (17.4%)	10 (20.0%)	
C1-2 *n* (%)	28 (28.6%)	13 (26.0%)	
Duration of follow-up (years)	4.19 ± 3.05	2.91 ± 2.46	*p* < 0.05
Proteinuria at FU (mg/kg/d)	0.0 (0–370)	0.0 (0–84)	NS
Creatinine at FU (mg/dL)	0.71 ± 0.21	0.7 ± 0.16	NS
GFR at FU (mL/min)	101.0 ± 24.45	100.62 ± 20.13	NS
Treatment:			
ACEI/ARB/none	59.2% (*n* = 58)	42.0% (*n* = 21)	*p* < 0.05
Glucocorticosteroids alone	13.3% (*n* = 13)	22.0% (*n* = 11)	NS
Immunosuppression + glucocorticosteroids	27.6% (*n* = 27)	34.0% (*n* = 17)	NS

ACEI—angiotensin-converting enzyme inhibitor; ARB—angiotensin receptor blocker; FU—end of follow-up; *n*—number of patients.

**Table 3:** Clinical characteristics of the patient groups based on the MEST-C score.

	Group I MEST-C ≤ 1	Group II MEST-C > 1	*p*
Age at diagnosis (years)	11.05 ± 4.14	12.52 ± 4.15	NS
Proteinuria at baseline (mg/kg/d)	12.8 (0–920)	16.4 (0–967)	NS
Creatinine at baseline (mg/dL)	0.64 ± 0.18	0.84 ± 0.42	*p* < 0.001
GFR at baseline (mL/min)	104.96 ± 37.32	86.5 ± 30.63	*p* < 0.01
Albumin (mg/dL)	3.99 ± 0.69	3.72 ± 0.91	NS
IgA (mg/dL)	251.04 ± 125.97	309.61 ± 139.45	*p* < 0.01
C3 (mg/dL)	122.83 ± 30.43	116.58 ± 28.3	NS
C4 (mg/dL)	23.21 ± 7.54	24.78 ± 9.1	NS
Intensity of IgA deposits (*n*%)			NS
+1	18 (24.3%)	25 (35.7%)	
+2	15 (20.3%)	14 (20.0%)	
+3	22 (29.7%)	21 (30.0%)	
+4	19 (25.7%)	10 (14.3%)	
Duration of follow-up (years)	4.27 ± 3.21	3.41 ± 2.53	NS
Proteinuria at FU (mg/kg/d)	0.0 (0–68)	0.0 (0–97)	*p* < 0.05
Creatinine at FU (mg/dL)	0.73 ± 0.19	0.71 ± 0.22	NS
GFR at FU (mL/min)	97.98 ± 20.72	99.37 ± 22.42	NS
Albumin (mg/dL)	4.37 ± 0.47	4.32 ± 0.36	NS
IgA (mg/dL)	248 ± 102.34	285.16 ± 138.75	NS
C3 (mg/dL)	106.13 ± 24.71	105.32 ± 25.59	NS
C4 (mg/dL)	19.86 ± 6.35	25.21 ± 18.97	NS

FU—end of follow-up.

**Table 4:** Clinical characteristics of the patient groups based on serum C3 level.

	Group 1 (*n* = 13) C3 below Reference Range	Group 2 (*n* = 135) C3 within Reference Range	*p*
Age at diagnosis (years)	10.97 ± 3.95	11.65 ± 4.29	NS
Proteinuria at baseline (mg/kg/d)	15.0 (0–967)	14.46 (0–920)	NS
Creatinine at baseline (mg/dL)	0.61 ± 0.22	0.76 ± 0.35	NS, *p* = 0.06
GFR at baseline (mL/min)	104.73 ± 43.32	95.54 ± 31.71	
Albumin (mg/dL)	3.99 ± 0.69	3.72 ± 0.91	NS
IgA (mg/dL)	232.13 ± 64.38	282.2 ± 140.54	NS
C4 (mg/dL)	18.27 ± 4.43	24.07 ± 8.37	*p* = 0.01
Intensity of IgA deposits (*n*%)			NS
+1	31 (26.0%)	3 (23.0%)	
+2	24 (20.2%)	4 (30.8%)	
+3	38 (31.9%)	3 (23.1%)	
+4	26 (21.9%)	3 (23.1%)	
Duration of follow-up (years)	4.11 ± 2.57	3.63 ± 2.94	NS
Proteinuria at FU (mg/kg/d)	0.0 (0–11.4)	0.0 (0–370)	NS
Creatinine at FU (mg/dL)	0.63 ± 0.11	0.72 ± 0.22	NS
GFR at FU (mL/min)	109.57 ± 14.89	100.13 ± 23.0	NS, *p* = 0.07
Albumin (mg/dL)	4.21 ± 0.36	4.33 ± 0.44	NS
IgA (mg/dL)	206.59 ± 60.0	264.63 ± 129.37	NS
C4 (mg/dL)	23.8 ± 17.58	21.95 ± 14.26	NS

FU—end of follow-up.

## Data Availability

The data analyzed in this study are available from the corresponding author on reasonable request.
